# Development of a dynamic Chest Wall and operating table simulator to enhance congenital heart surgery simulation

**DOI:** 10.1186/s41205-020-00067-4

**Published:** 2020-06-01

**Authors:** Brandon Peel, Pascal Voyer-Nguyen, Osami Honjo, Shi-Joon Yoo, Nabil Hussein

**Affiliations:** 1 Center for Image-Guided Innovation and Therapeutic Intervention (CIGITI), Hospital for Sick Children, University of Toronto, Toronto, Ontario Canada; 2 Division of Cardiology, Department of Paediatrics and Division of Cardiovascular Surgery, Department of Surgery, Hospital for Sick Children, University of Toronto, 555 University Avenue, Toronto, Ontario M5G1X8 Canada; 3 Department of Diagnostic Imaging and Division of Cardiology, Department of Paediatrics Hospital for Sick Children, University of Toronto, Toronto, Ontario Canada

**Keywords:** Congenital heart surgery, Surgical simulation, 3D-printing, Chest wall simulator

## Abstract

**Background:**

The Hands-On Surgical Training in Congenital Heart Surgery (HOST-CHS) program using 3D printed heart models has received positive feedback from attendees. However, improvements were necessary in the simulator set up to replicate the ergonomics experienced in the operating room. This paper illustrates the development of a dynamic chest wall and operating table simulator to enhance the simulation experience.

**Methods:**

The simulator was designed to address the limitations with the existing set up. This included a suboptimal operating position, unrealistic surgical exposure and limitations in illuminating the operative field and recording procedures. A combination of computer-aided design and various 3D-printing techniques were used to build the components. The simulator’s usefulness was evaluated by surgeons who attended the 5th annual HOST course via a questionnaire.

**Results:**

The simulator consists of three components; an operating table simulator which allows height adjustment and pitch-and-roll motion; a suture retraction disc, which holds sutures under tension to improve exposure; and a pediatric chest wall cavity to replicate a surgeon’s access experience during surgery. Nineteen surgeons completed the questionnaire. All surgeons agreed that the addition of the simulator was acceptable for surgical simulation and that it helped replicate the ergonomics experienced in the operating room.

**Conclusions:**

The inclusion of the HOST-CHS simulator adds value to simulation in congenital heart surgery (CHS) as it replicates the view and exposure a surgeon experiences. Improvements like these will help develop high-fidelity simulation programs in CHS, which could be utilized to train surgeons globally.

## Introduction

Simulation is emerging as a tool to compliment surgical training and skill acquisition in congenital heart surgery (CHS), however its widespread use is limited [1–5]. The Hands-On Surgical Training in Congenital Heart Surgery (HOST-CHS) program uses 3D-printed heart models to simulate the technically complex operations required to treat congenital heart diseases. Despite the positive feedback from attendee surgeons improvements were necessary in the simulator set up to replicate the ergonomics experienced in the operating room [1, 2].

### Limitations of HOST-CHS set up

**Fig. 1:**
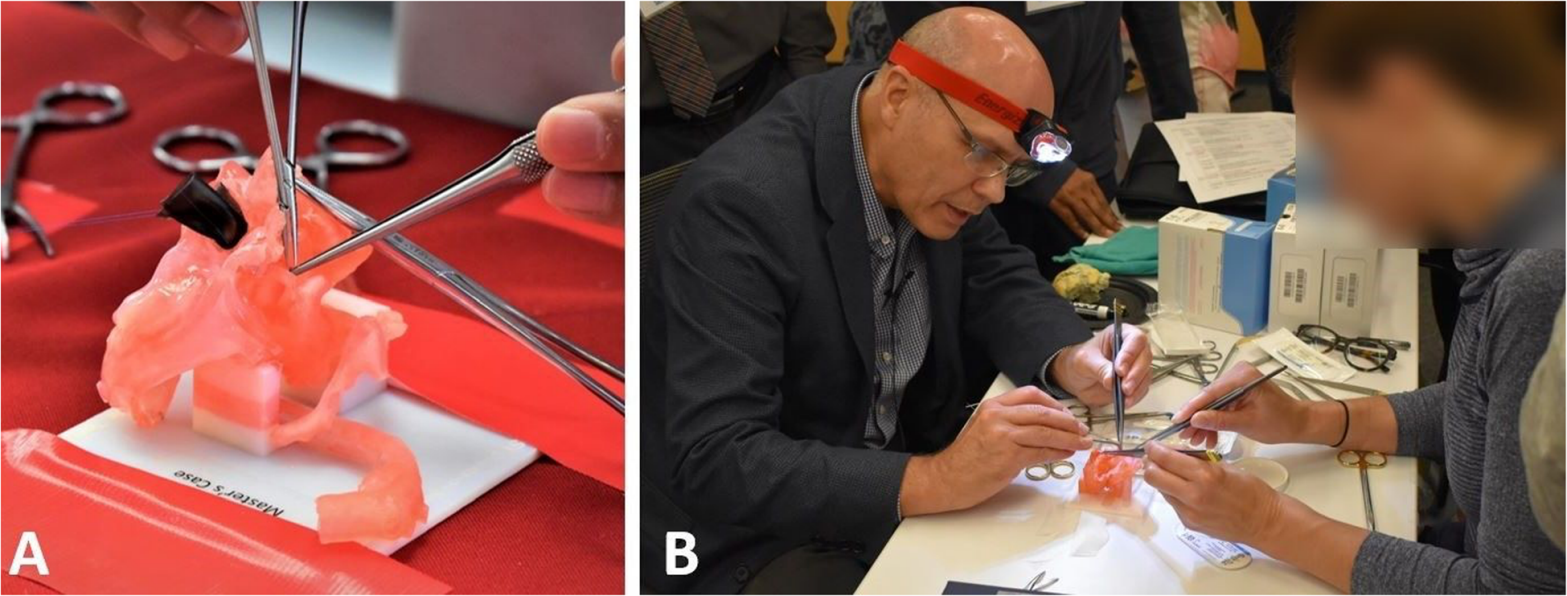
**a** A surgeon simulating a complex congenital heart surgical procedure on a 3D-printed heart model at the hands-on surgical training (HOST) course. **b** A congenital heart surgeon demonstrating the arterial switch operation on a 3D-printed heart model at the HOST course. Note that the models are stuck to the table at a fixed height forcing the surgeon to sit down preventing them from using their surgical loupes. A simple headlight is used to illuminate the model. The procedure is video recorded for retrospective assessment (not shown)

Four aspects were identified as limitations of the original set up for the HOST-CHS course (Fig. 1):

1. Suboptimal operating position – 3D-printed heart models were fixed to the table with tape limiting operating height. Subsequently surgeons were prevented from operating at the ideal height. Models were also unable to be rotated to improve exposure of the operative field.
2. Unrealistic surgical exposure – The absence of a chest wall allowed surgeons to operate from any angle, when in reality surgeons operate within a fixed region (i.e. median sternotomy incision).
3. Limitations in light exposure – Although commercially available headlights were used, the battery life limited the duration of the light’s effectiveness during simulation.
4. Difficulties in video-recording for accurate objective assessment

This paper illustrates the development of a dynamic chest wall and operating table simulator to enhance the simulation experience.

### Components for the Hands-On Surgical Training-Congenital Heart Surgery (HOST-CHS) simulator (Fig. 2)

#### Operating table simulator

A scissor-lift (LBJSET - United Scientific Supplies Inc., Waukegan, IL, USA) forms the lowest aspect of the simulator and has a dial mechanism to adjust the height of the models. Lighting and recording devices are attached to the top platform to allow surgical procedures to be recorded (Fig. 3). Roll and pitch components were designed on SolidWorks™ (Dassault Systèmes SolidWorks, Concord, MA) and 3D-printed on the Objet 500 Connex3 (Stratasys, Eden Prairie, MN) using VeroWhitePlus resin material and is attached to the scissor lift. This design utilizes perpendicular hinges to allow 30-degree angle rotation in all directions. Clamping handles are used to lock the chest simulator in the desired position and are easily adjustable.

**Fig. 2:**
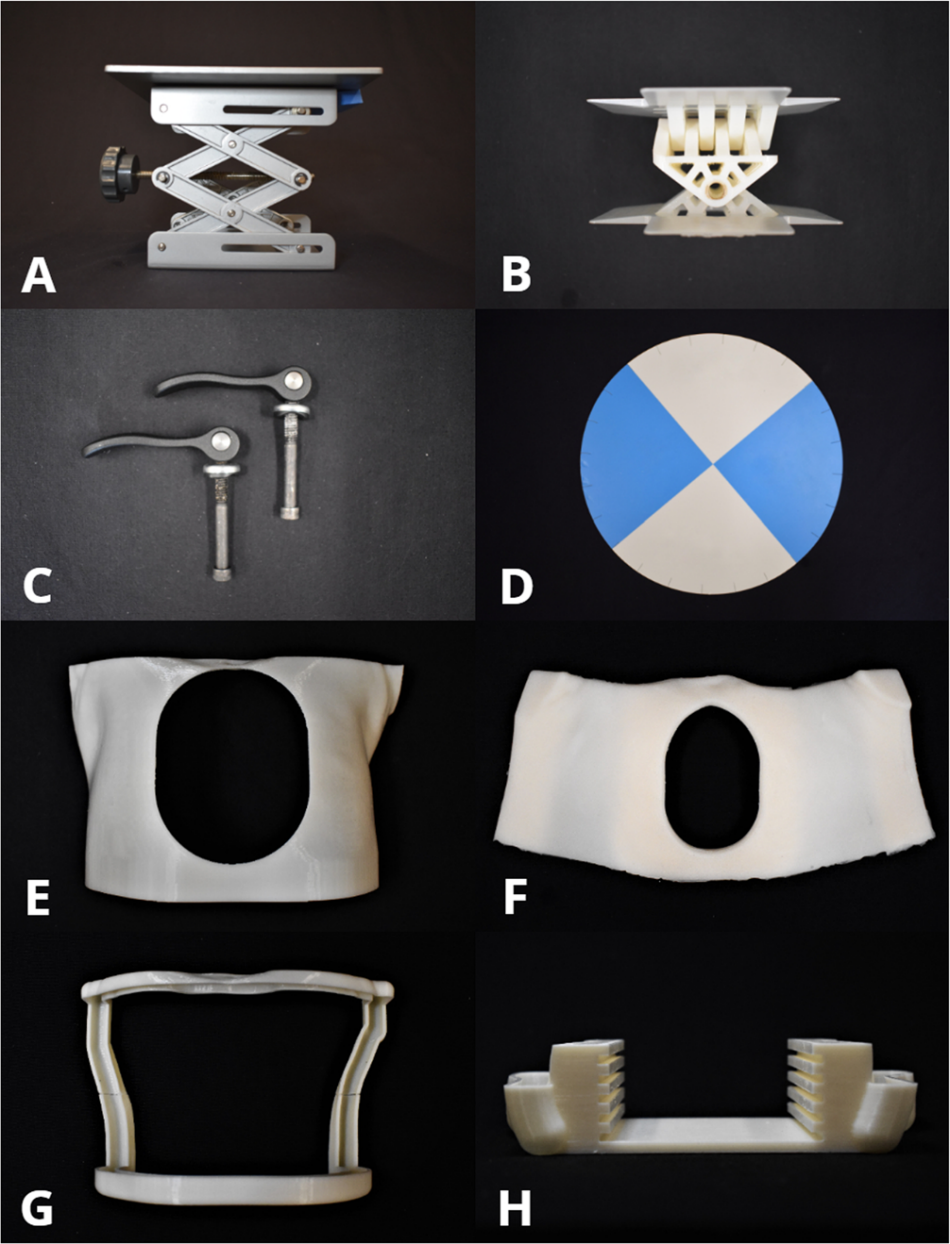
The components used for the Hands-On Surgical Training - Congenital Heart Surgery (HOST-CHS) simulator. **a**: Commercially available scissor lift (LBJSET - United Scientific Supplies Inc., Waukegan, IL, USA). **b**: Roll and pitch 3D-printed components. **c**: Commercially available cam handles with internal thread (McMaster Carr, Inc., Cleveland, OH). **d**: Suture retraction disk. **e**: Chest wall with median sternotomy incision, **f**: Silicone skin, **g**: Upper and lower brackets which secure the silicone skin in place, **h**: Lower holder with notches at five different levels to fit all 3D printed heart models used during the HOST course

**Fig. 3:**
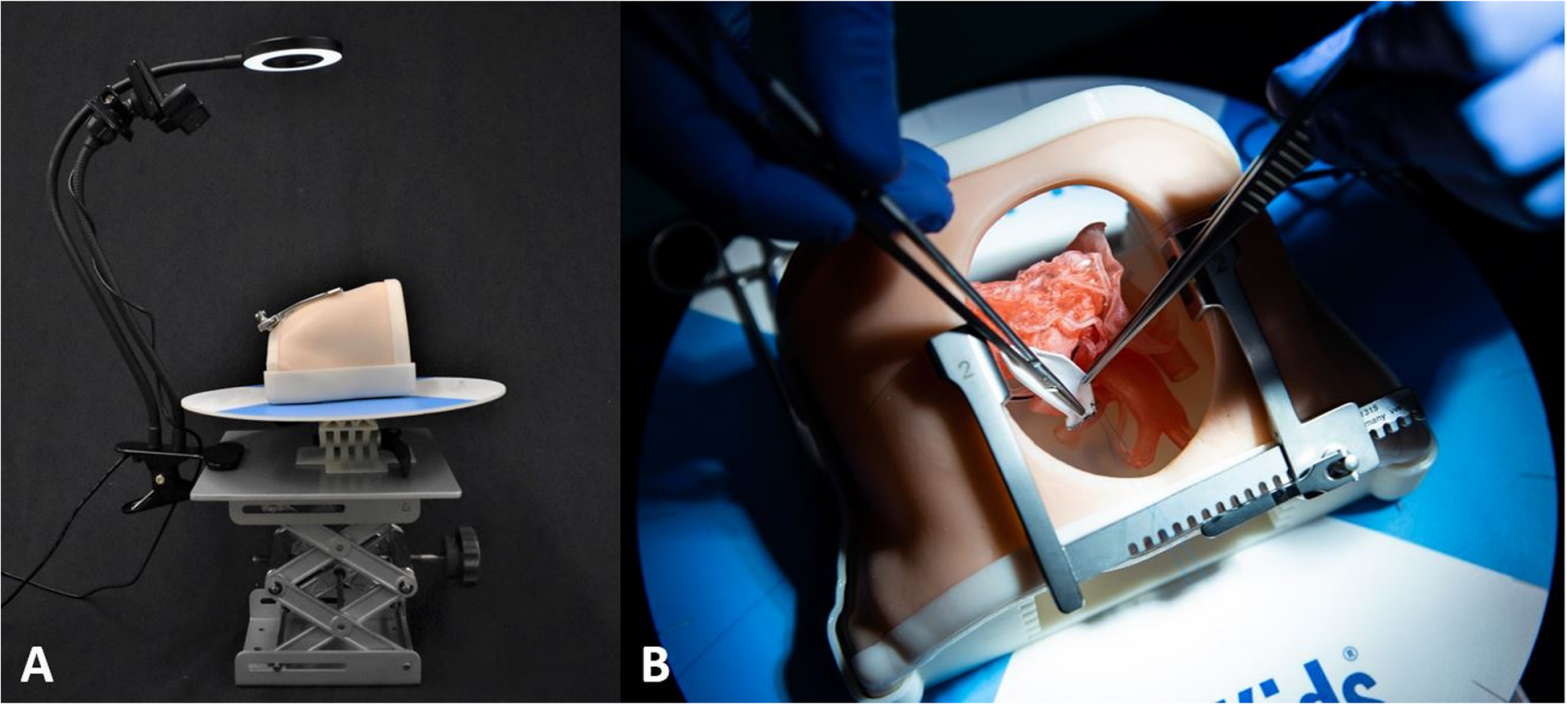
**a**: The complete assembly up of the Hands On Surgical Training in Congenital Heart Surgery (HOST-CHS) simulator. This assembly includes the pediatric chest wall cavity, suture retraction disk, roll and pitch components, operating table simulator, a webcam and lighting equipment. **b** A surgeon suturing a transannular patch on a tetralogy of Fallot 3D-printed heart model through the sternotomy incision of the HOST-CHS simulator. The 3D-printed heart model has been placed at the optimized height inside the holder, while the surgeon utilizes the suture retraction disk

#### Suture retraction disk

During a surgical procedure, the surgeon needs the ability for sutures to be held securely, whilst they focus on another aspect of the operation. A suture retraction disc was designed with 24 equally spaced slits around the circumference to allow sutures of different sizes to be held securely in place and is attached to the top of the pitch and roll mechanism.

#### Pediatric Chest Wall cavity

An anatomically accurate pediatric chest wall cavity was designed and 3D-printed to reproduce the surgeon’s access experienced during CHS. Dimensions were retrieved by computer-tomography images. Silicon was molded around the cavity to simulate patients’ skin (Dragon Skin 20 - Smooth-On Inc., Easton, PA). A lower holder completed the simulator with 5 various height levels, designed to fit all the 3D-printed heart models used during the HOST program. These components were 3D-printed on the Fortus380mc (Stratasys, Eden Prairie, MN) printer using ABS M30i filament. Video 1 demonstrates the assembly, functions and use of the simulator.


**Additional file 1 Video 1:** Demonstration of the chest wall simulator being assembled, the degrees of motion, suture retraction and a surgeon performing a transannular patch reconstruction as part of a tetralogy of Fallot repair on a 3D-printed heart model.


**Fig. 4:**
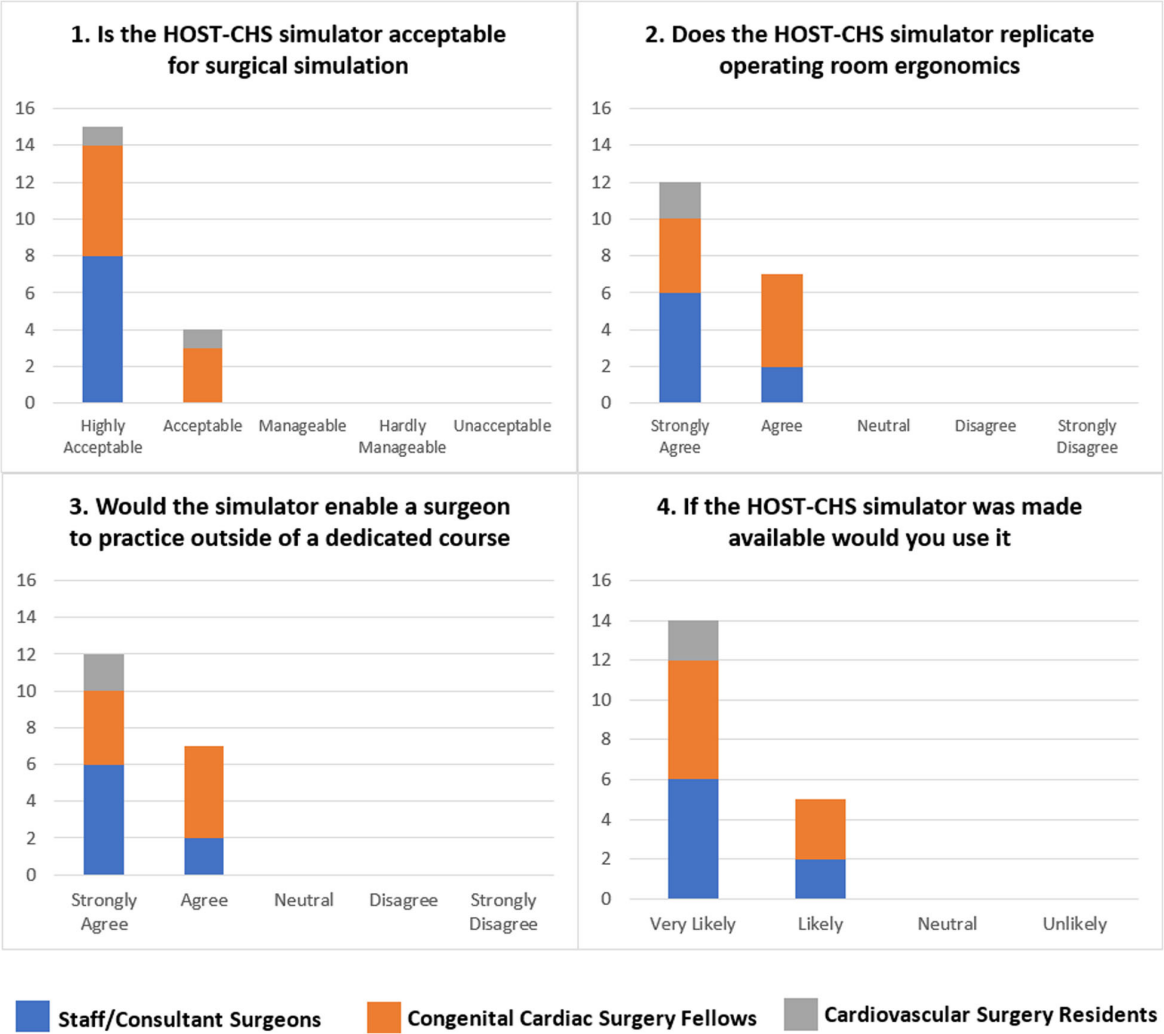
Questionnaire responses on the usefulness of the Hands-On Surgical Training in Congenital Heart Surgery (HOST-CHS) simulator by the congenital heart surgeons that participated in the 5th annual HOST course

The simulator was first trialled at the annual HOST course of the authors’ institution with 19 cardiovascular surgeons participating. Surgeons’ experience in CHS varied from resident surgeons to staff/consultant level. All surgeons agreed that the addition of the simulator was acceptable for surgical simulation and that it helped replicate the surgical ergonomics. All agreed that the simulator would encourage practice outside of a dedicated course and would be keen to use it more if made available (Fig. 4).

## Discussion

With the growing expectation of perfect patient outcomes, there is a need for improved training methods by implementing simulation in CHS [2]. By producing a simulator that can be used in any environment, surgeons are able to rehearse technically challenging procedures in inconsequential environments. Simulators have been shown to be effective in training and have been successfully incorporated into national training programs in adult cardiothoracic surgery [6–8]. However, within CHS there is a clear gap in simulation, where one could argue the need is greater due to the high stakes involved and the complexity/limitation of cases [2].

The development of the HOST-CHS simulator was successful in addressing the requirements set by the surgeons’ recommendations. The operating table component allowed the surgeon to set a personalized height and control the roll, pitch and tilting motions to reproduce the real ergonomics. The suture retraction disk improved surgical exposure and the overall design allowed for the inclusion of lighting and recording equipment. The chest wall component restricted the surgeons approach to the heart model, which replicates reality.

The feedback from the participant surgeons was encouraging with all grades of surgeon agreeing that the inclusion of the simulator was acceptable for the simulation of CHS and that it helps replicate the ergonomics experienced in the operating room. All surgeons agreed that the simulator would encourage simulation outside of dedicated courses and were likely to incorporate it into their own institution if made available. The number of participants who completed this questionnaire may be a limitation, however the overall consensus from this study strongly suggests that the incorporation of the HOST-CHS chest simulator as part of the HOST program is beneficial to the overall simulation experience.

## Future directions

**Fig. 5:**
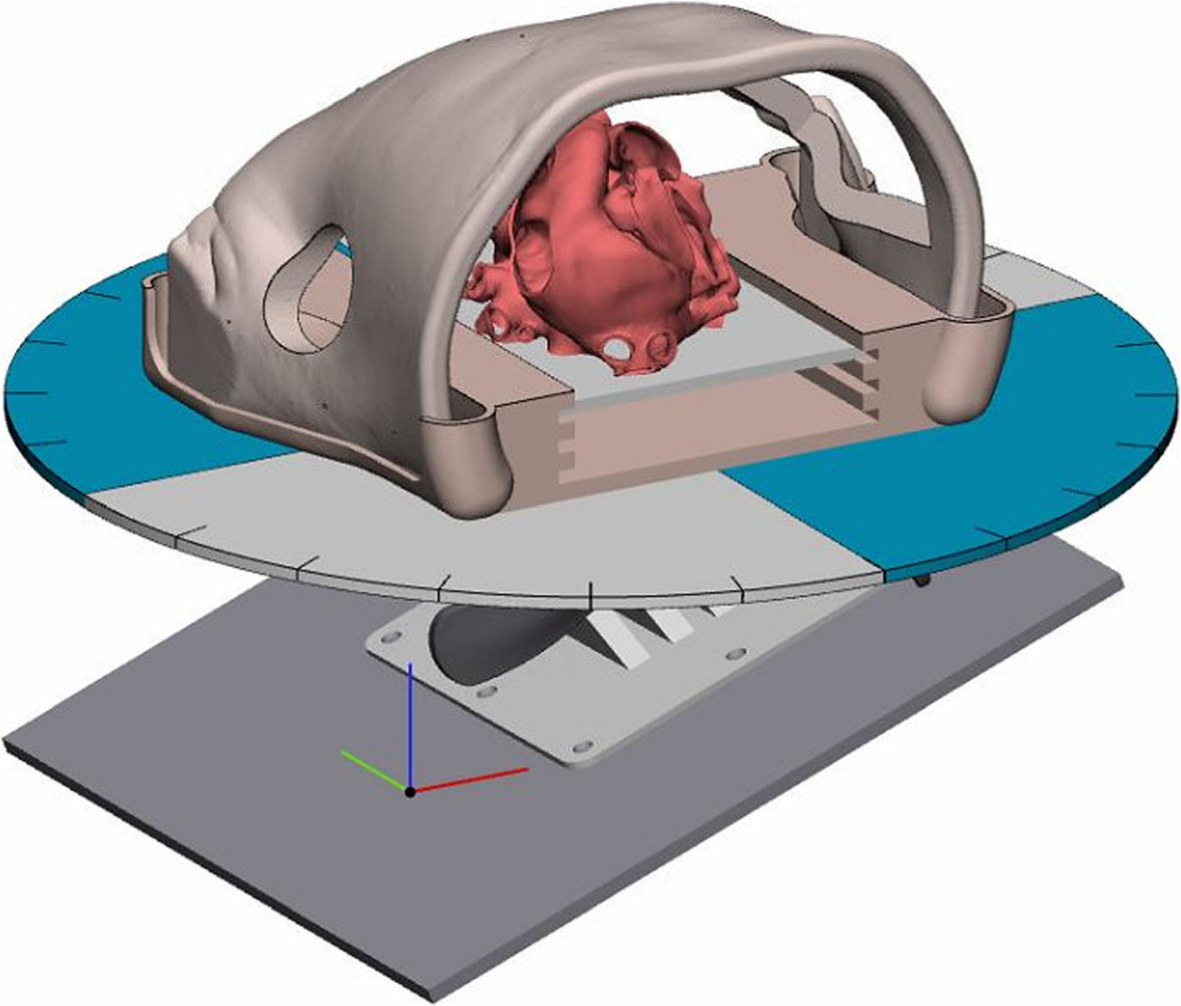
Computer render of a minimally invasive dynamic chest wall and operating table simulator. Note the absence of a median sternotomy incision and the inclusion of a mini-thoracotomy incision the surgeon will operate through. The opposite side of the chest is removed to allow the teacher to observe the performance of the surgeon

With the increasing trend towards minimally invasive surgery, simulators will be required to validate and improve methods prior to real-life surgery. Within our institution, we are developing a chest wall simulator designed specifically for minimally-invasive congenital heart surgery (Fig. 5). This simulator will allow staff surgeons to rehearse operations and teach new techniques, which will be increasingly difficult to do in reality. Current limitations in the 3D-printing techniques and materials limits the inclusion of flow circuits with 3D-printed heart models, however there have been attempts to overcome this making it a tangible reality [2]. It is expected that with the ongoing improvements in print materials, this is the next step in simulation.

## Conclusion

The inclusion of the HOST-CHS simulator adds value to simulation in congenital heart surgery as it replicates the view and exposure a surgeon experiences. With training limitations being a global problem for congenital heart surgeons it is expected that simulators like these will be increasingly utilized in surgical training.

## Data Availability

All data generated or analysed during this study are included in this published article.

## Abbreviations

HOST-CHS: Hands-on surgical training in congenital heart surgery
CHS: Congenital heart surgery
HOST: Hands-on surgical training
3D: Three-dimensional
